# Medical and Philosophical News

**Published:** 1781-04

**Authors:** 


Mr. Thomas Henry of Mancheſter, F. R. S. is about to publiſh a work, entitled, "An Ac-

"count of a cheap and eaſy method of pre-

"ſerving Water at ſea from Putrefaction, with-

" out

"out injury to its original pleafantnefs and pu-
"rity: to which is added, a mode of impreg-
"nating water in large quantities with fixed
"air for medicinal ufes, on fhipboard and in
"hofpitals; and likewife a procefs for the pre-
"paration of artificial yeaft."

